# *Tuwongella immobilis* gen. nov., sp. nov., a novel non-motile bacterium within the phylum *Planctomycetes*

**DOI:** 10.1099/ijsem.0.002271

**Published:** 2017-10-31

**Authors:** Christian Seeger, Margaret K. Butler, Benjamin Yee, Mayank Mahajan, John A. Fuerst, Siv G. E. Andersson

**Affiliations:** ^1^​ Department of Cell and Molecular Biology, Molecular Evolution, Uppsala University, Box 596, 751 24 Uppsala, Sweden; ^2^​ Australian Center for Ecogenomics, School of Chemistry and Molecular Biosciences, University of Queensland, Brisbane, Queensland 4072, Australia; ^3^​ School of Chemistry and Molecular Biosciences, University of Queensland, Brisbane, Queensland 4072, Australia

**Keywords:** *Planctomycetes*, freshwater bacteria, nucleoid, phylogeny, fatty acids

## Abstract

A gram-negative, budding, catalase negative, oxidase positive and non-motile bacterium (MBLW1^T^) with a complex endomembrane system has been isolated from a freshwater lake in southeast Queensland, Australia. Phylogeny based on 16S rRNA gene sequence analysis places the strain within the family *Planctomycetaceae*, related to *Zavarzinella formosa* (93.3 %), *Telmatocola sphagniphila* (93.3 %) and *Gemmata obscuriglobus* (91.9 %). Phenotypic and chemotaxonomic analysis demonstrates considerable differences to the type strains of the related genera. MBLW1^T^ displays modest salt tolerance and grows optimally at pH values of 7.5–8.0 and at temperatures of 32–36 °C. Transmission electron microscopy analysis demonstrates the presence of a complex endomembrane system, however, without the typically condensed nucleoid structure found in related genera. The major fatty acids are 16 : 1 ω5c, 16 : 0 and 18 : 0. Based on discriminatory results from 16S rRNA gene sequence analysis, phenotypic, biochemical and chemotaxonomic analysis, MBLW1^T^ should be considered as a new genus and species, for which the name *Tuwongella immobilis* gen. nov., sp. nov. is proposed. The type strain is MBLW1^T^ (=CCUG 69661^T^=DSM 105045^T^).

The family *Planctomycetaceae* belongs to the phylum *Planctomycetes* within the domain Bacteria, members of which possess distinctive properties such as a complex endomembrane system, budding reproduction and the ability to perform endocytosis-like protein uptake [1, 2]. The family *Planctomycetaceae* is formed by the described genera *Blastopirellula, Gemmata, Gimesia, Pirellul*a*, Planctomicrobium*, *Planctopirus, Rhodopirellula, Rubinisphaera, Schlesneria, Telmatocola, Thermogutta* and *Zavarzinella* [3–11]. Previously included in the family were the genera of *Isosphaera*, *Singulisphaera*, *Aquisphaera* and *Paludisphaera*, but these genera have recently been re-classified under the family *Isosphaeraceae* [12]. Here, we describe an isolate from a freshwater lake that is closely related to members of the genera *Gemmata*, *Telmatocola* and *Zavarzinella* but has a number of distinctive molecular and phenotypic features that distinguish it from the respective type strains *G. obscuriglobus*, *T. sphagniphila* and *Z. formosa*.

Strain MBLW1^T^ was isolated from water samples collected from the freshwater University Lake at the University of Queensland, Brisbane, Australia. Samples were collected, using sterile bottles, from under the water surface at a level no deeper than 30 cm. Water samples were concentrated via filtration through a 0.45 µm membrane filter, with the concentrated particulate material on the filter being resuspended in approximately 3 ml of sterile lake water filtrate. Lake Water (LW) agar plates (15 g agar autoclaved in 250 ml distilled water, upon cooling 25 µg ml^−1^ filter-sterilised ampicillin added and the volume made up to 1 l with the sterile filtrate that resulted above) were spread with 100 µl of the water concentrate. Plates were then incubated in the light at room temperature in a CO_2_-enriched atmosphere generated in an anaerobic jar containing an Alka-seltzer dissolved in a beaker of water [13]. After 38 days, colonies were subcultured to purity and maintained on M1 agar [14], in the dark, at 28 °C, aerobically. During the following cultivation on LW agar plates, the incubation time decreased to approximately 5 days at 28 °C. MBLW1^T^ was subsequently cultivated aerobically in the dark on M1 agar plates at 28 °C. Colonies of MBLW1^T^ were smooth, with an entire edge, translucent pink in colour, up to 4 mm in diameter and had a large, flat zone of growth with a slightly raised centre, giving them a ‘fried egg’ appearance.

16S rRNA gene sequence of MBLW1^T^ was determined by PCR and sequencing. The phylogenetic analyses of MBLW1^T^ were based on alignments of 16S rRNA sequences from 25 *Planctomycetes* species. The 16S rRNA gene sequences were aligned using silva Incremental Aligner [15] and the hyper-variable regions were removed from the aligned sequences using the Lane mask [16]. The phylogeny was performed in RaxML version 8.0.26 using the maximum likelihood method with 50 bootstraps and the GTRGAMMAI nucleotide substitution model [17].

The strain MBLW1^T^ shared 91.9 % sequence similarity with *G. obscuriglobus* which makes it a new sister genus to *Gemmata*. Pairwise sequence similarity measures among cultivated species showed that MBLW1^T^ was most similar to both *Z. formosa* and *T. sphagniphila*, with a sequence similarity of 93.5 and 93.3 % respectively. The maximum likelihood phylogeny confirmed that MBLW1^T^ clusters with *G. obscuriglobus*, *Z. formosa* and *T. sphagniphila* with 100 % bootstrap support, although their internal diversification pattern could not be resolved with significant support solely by 16S rRNA gene phylogeny (Fig. 1). The DNA G+C content of MBLW1^T^ was determined to be 57 mol%, compared to 67 mol% in *G. obscuriglobus* and 59 mol% in both *T. sphagniphila* and *Z. formosa.* The DNA G+C content information for strain MBLW1^T^ was calculated from the genome sequence (Mahajan, Yee, Fuerst, Andersson, unpublished data).

**Fig. 1.**
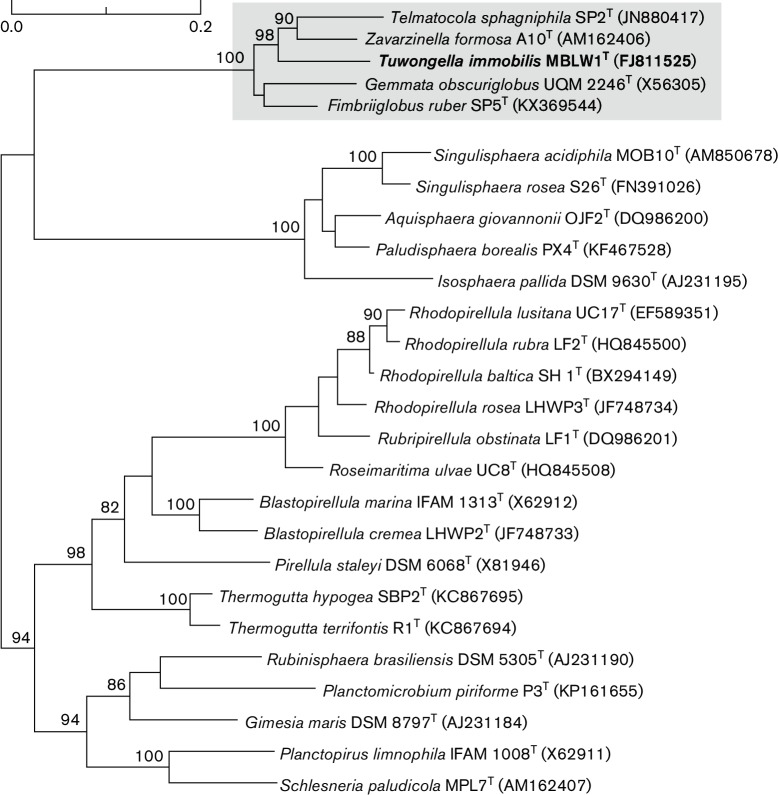
Phylogenetic placement of MBWL1^T^ within the order of *Planctomycetales.* The phylogeny was inferred from a 16S rRNA gene alignment using the maximum likelihood method. Numbers on the nodes refer to bootstrap support values. Only bootstrap support values higher than 75 % are shown. Nucleotide sequence accession numbers are presented next to the species names.

The 16S rRNA gene sequence of strain MBLW1^T^ possess all *Planctomycetes* signature nucleotides [18], except for the nucleotide in position 983 : 1, which is missing. The MBLW1^T^ 16S rRNA also possess all *Gemmata* group signature nucleotides [19], except at positions 668 : 738 (U : A instead of the signature C : G), 680 : 710 (G : U instead of the signature C : G/U : A) and 1420 : 1480 (G : C instead of the signature A : U) (*Escherichia coli* numbering) [20]. Interestingly, in distinction to *Z. formosa*, *G. obscuriglobus* and all other *Gemmata* clade sequences, the MBLW1^T^16S rDNA sequence lacks the 10-base indel between *E. coli* positions 998 and 999, which is otherwise characteristic of the *Gemmata* group [21], including *Z. formosa* [5] and have an extended helix, Helix 10, compared to other members of the *Gemmata* group.

The majority of the phenotypic, biochemical and chemotaxonomic analyses were performed with MBLW1^T^ and *G. obscuriglobus* ACM 2246^T^. *Z. formosa* DSM 19928^T^ was only included for TEM analysis. Both *T. sphagniphila* and Z*. formosa* require cultivation under acidic conditions and at relatively low temperatures (20–25 °C) [5, 6], while MBLW1^T^ and *G. obscuriglobus* could be cultivated under comparable conditions on M1 agar plates, at neutral pH and elevated temperature (>30 °C). Furthermore, both *T. sphagniphila* and *Z. formosa* grow considerably slower than MBLW1^T^ and *G. obscuriglobus*.

For analysis by transmission electron microscopy (TEM), MBLW1^T^ and *G. obscuriglobus* were cultivated for 4 days at 28 °C on M1 agar plates. *Z. formosa* was cultivated on DSM 1196 agar plates for 30 days at 24 °C. Cells were fixed for 15 min at room temperature in 2 % glutaraldehyde, 1 % paraformaldehyde in 0.01 M phosphate buffer, pH 7.4. The cells were further fixed and stored at 4 °C. Further preparation of the specimen was performed by the electron microscopy unit, Emil, at Karolinska Institutet, Huddinge, Sweden. After fixation, cells were rinsed in 0.01 M phosphate buffer and centrifuged. The pellets were then postfixed in 2 % osmium tetroxide (TAAB) in 0.01 M phosphate buffer, pH 7.4 at 4 °C for 2 h, dehydrated in ethanol, followed by acetone and embedded in LX-112 (Ladd Research). Ultrathin sections (~50–60 nm) were cut by a Leica EM UC 6. Sections were contrasted with uranyl acetate followed by lead citrate and examined in a Hitachi HT 7700 at 80 kV. Digital images were taken by using a Veleta camera (Olympus Soft Imaging Solutions). For negative staining, MBLW1^T^ was grown on M1 agar plates at 28 °C for 2 days, and a suspension of the cells was stained with 1.0 % uranyl acetate in 0.4 % (w/v) sucrose. Cells were examined via a JEOL 1010 transmission electron microscope. For phase contrast microscopy, MBLW1^T^ and *G. obscuriglobus* were grown for 4 days on M1 agar plates at 28 °C, resuspended in M1 medium and a drop from the cell suspension was placed on a slab of 1 % agarose (w/v) on a slide and allowed to soak in. Cover slips were placed over the agarose and samples viewed using a Zeiss Axioplan2 fluorescence microscope. Motility was checked by phase contrast microscopy of a suspension of cells placed directly onto a slide. For this, a preparation of cells from just-visible colonies growing on an M1 agar plate was used.

Phase-contrast images of MBLW1^T^ show phase-variable regions within the cells and TEM analysis revealed a complex endomembrane system, similar to *G. obscuriglobus* and *Z. formosa* (Fig. 2). Compared to *G. obscuriglobus* and *Z. formosa* that both display very distinct nucleoid structures, the nucleoid of MBLW1^T^ is only weakly visible in some TEM micrographs. Crateriform structures, a defining feature within the *Planctomycetes* phylum [1], were visible over the entire surface of negatively stained MBLW1^T^ cells. Fimbriae could also be observed. Motility of MBLW1^T^ has not been observed, not even in very young cultures and this is consistent with the lack of flagella observed in negatively stained cells (Fig. S1a, available in the online version of this article). This is in contrast to *G. obscuriglobus* and other *Gemmata*-like species, like *Z. formosa*, all of which are motile at some stage of the life cycle [3, 5, 21]. No stalks were apparent in MBLW1^T^ when observed via phase contrast microscopy or TEM of negatively stained cells (Fig. S1b). Mature cells were cocci, with the cell diameter of MBLW1^T^ ranging from 2.2 to 3.1 µm. MBLW1^T^ reproduced by budding. No rosette or chain formation of the cells was observed.

**Fig. 2.**
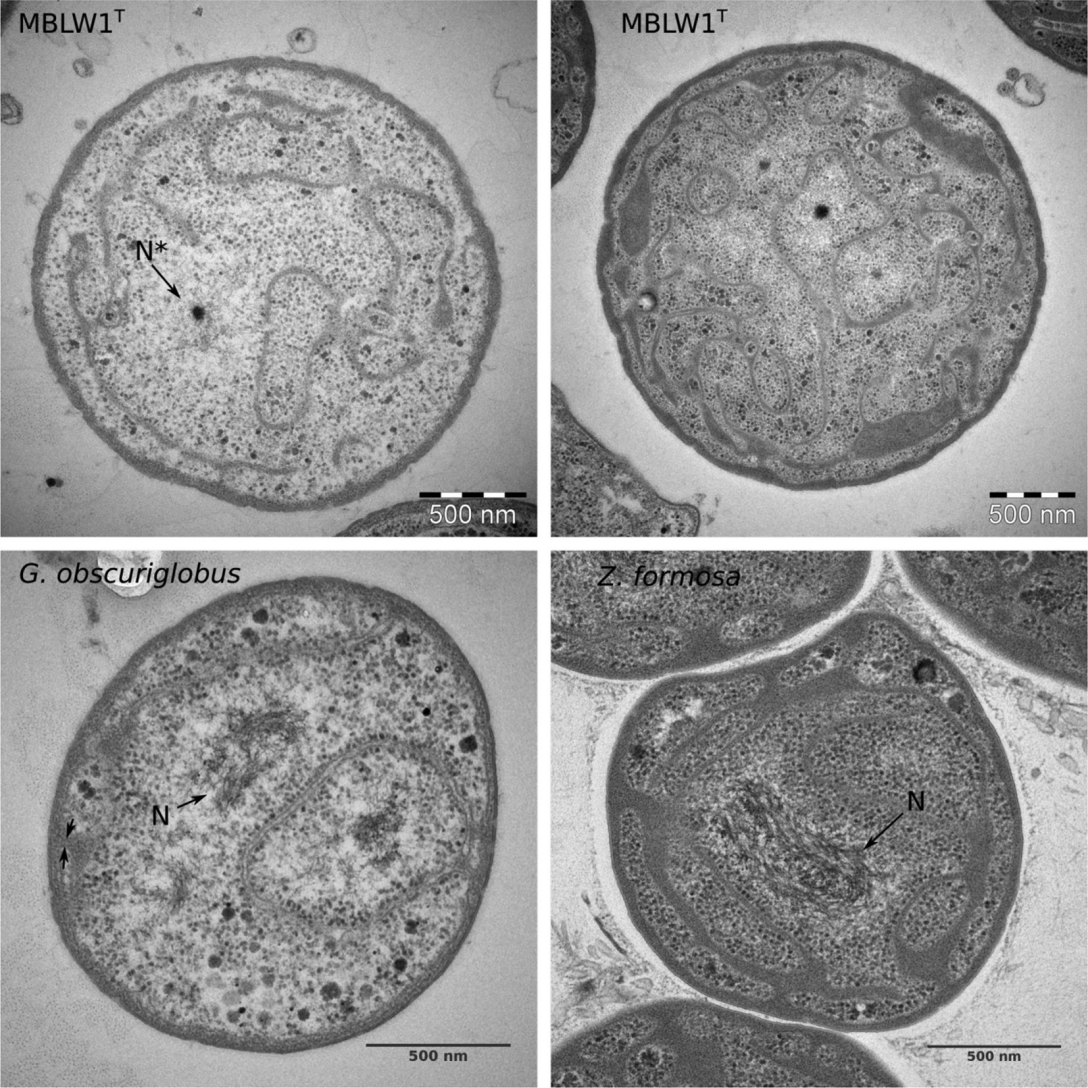
Transmission electron micrographs show the complex endomembrane system in MBLW1^T^, *G. obscuriglobus* and *Z. formosa*, a distinct nucleoid (N) in *G. obscuriglobus* and *Z. formosa* and diffuse fibrillar nucleoid structures (N*) in one of the two MBWL1^T^ cells.

The growth conditions of MBLW1^T^ were investigated on M1 agar plates and in M1 liquid medium. On M1 agar plates, MBLW1^T^ was able to grow at temperatures of 20 to 40 °C. In M1 liquid medium, growth of MBLW1^T^ was monitored by turbidometry at a wavelength of λ=600 nm and the optimal growth temperature was found between 32 and 36 °C (Fig. S2a) with corresponding generation times of g_32_ ~11 h and g_36_ ~6 h, respectively. Growth of MBLW1^T^ under different pH conditions was investigated in M1 liquid media and was performed to distinguish MBLW1^T^ from the acidophilic species *Z. formosa* and *T. sphagniphila*, and establish the optimum pH for growth. Variations of the pH between pH 6.0–8.0 were achieved by using a Na_2_HPO_4_-NaH_2_PO_4_ buffering system, variations from pH 9.0–10.5 were achieved by using a Na_2_CO_3_-NaHCO_3_ buffering system. MBLW1^T^ was grown for 48 h at 32 °C in Nunc Edge 96-well microplates (Thermo Scientific) and the optical density (λ=600 nm) was measured using a Tecan Infinite M200 microplate reader (Tecan). Growth of MBLW1^T^ was observed from pH 6.0–10.5, optimal growth occurred at pH 7.5–8.0 (Fig. S2b). Salt tolerance of MBLW1^T^ and *G. obscuriglobus* was compared in 24-well M1 agar plates containing increasing NaCl-concentrations [0.0 %–2.0 % (w/v)] after incubation for 5 days at 32 °C. Growth of MBLW1^T^ was observed up to 0.5 % (w/v) NaCl, at concentrations between 0.6 % (w/v) and 0.8 % (w/v), the cells were only growing weakly. Growth of *G. obscuriglobus* was observed up to 1.5 % (w/v) NaCl but not observed at 2.0 % (w/v) NaCl. Additionally, MBLW1^T^ was grown in M1 liquid media in a 24-well plate format in the presence of increasing NaCl concentrations from 0.0–0.8 % (w/v). After incubation for 5 days at 30 °C, the optical density at λ=600 nm was measured in a UV-spectrophotometer and values of the NaCl-containing samples were compared with the NaCl-free samples to determine the relative growth. Growth of MBLW1^T^ was inhibited by more than 50 % at NaCl concentrations greater than 0.4 % (w/v) (Fig. S2c). At concentrations greater than 0.8 % (w/v) NaCl, essentially no growth of MBLW1^T^ was observed.

For comparing the antibiotic susceptibility of MBLW1^T^ and *G. obscuriglobus*, cells were grown on M1 agar plates (without CaCO_3_) for 7 days at 28 °C in the dark using discs containing ampicillin (10 µg), gentamicin (10 µg), kanamycin (30 µg), neomycin (10 µg) and streptomycin (10 µg). MBLW1^T^ and *G. obscuriglobus* are susceptible to kanamycin and neomycin and resistant to ampicillin and streptomycin. In contrast to *G. obscuriglobus*, MBLW1^T^ is resistant to gentamicin. Susceptibility of *G. obscuriglobus* to gentamicin is in accordance with previous results [22]. The comparison of antibiotic susceptibility profiles with *Z. formosa* and *T. sphagniphila* is shown in Table 1.

**Table 1.** Phenotypic characteristics of MBLW1^T^ in comparison to *G. obscuriglobus*, *T. sphagniphila* and *Z. formosa* The four strains utilize *N*-acetylglucosamine, glucose, galactose, maltose and rhamnose (weak growth in MBLW1^T^ and *G. obscuriglobus*). The four strains do not utilize fructose, mannitol and glycerol. MBLW1^T^ and *G. obscuriglobus* grow weakly on arabinose (not tested for *T. sphagniphila* and *Z. formosa*). The four strains are resistant to ampicillin and streptomycin but susceptible to kanamycin. R, resistant; S, susceptible.

Characteristic	MBLW1^T^	*G. obscuriglobus*	Z. formosa*	T. sphagniphila*
Cell shape	Spherical	Spherical to ovoid	Ellipsoidal	Spherical
Cell size (µm)	2.2–3.1	1.9–2.4	2.5–3.2 x 2.0–2.5	1.2–2.0
Rosette formation	−	−	+	+
Stalked	−	−	+	+
Motile	−	+	+	−
Growth pH <5.0	−	−*	+	+
Growth >30 °C	+	+	−	−
Major fatty acids	16 : 1ω5c, 16 : 0, 18 : 0	18 : 0, 16 : 1ω5c, 15 : 0 ANTEISO	18 : 1ω5c, 16 : 1ω5c, 18 : 0	16 : 1ω5c, 18 : 0, 18 : 1ω5c
DNA G+C content (mol%)	57	67†	59†	59
NaCl tolerance	0.5 %	1.5 %	0.6 %	0.1 %
Oxidase test	+	−	+	−
Catalase test	−	+	+	+
Urease test	+	−	−	−
Carbon sources				
Sucrose	−	+	+	+
Xylose	−	−	+	+
Antibiotic susceptibility				
Gentamicin	R	S	S	S
Neomycin	S	S	S	R

*Data based on Kulichevskaya *et al*. [5, 6].

†G+C contents based on Scheuner *et al*. [8].

The oxidase test based on the cytochrome oxidase catalysed reaction of N,N-dimethyl-p-phenylenediamine oxalate and α-naphthol to indophenol blue [23, 24] was performed using oxidase test discs (Sigma Aldrich). MBLW1^T^ and *G. obscuriglobus* were grown for 4 days at 28 °C on M1 agar plates and cells were transferred with a sterile cotton tip onto oxidase test discs. The species were deemed oxidase positive if the colour on the disc changed to deep purple blue within 2 min at room temperature. For the catalase test, 4 day-old cultures (M1 agar, 28 °C) of MBLW1^T^ and *G. obscuriglobus* were transferred with a sterile cotton tip into 1 ml 3 % (v/v) H_2_O_2_. Immediate formation of bubbles was indicative for a positive catalase test. For the urease test, MBLW1^T^ and *G. obscuriglobus* were grown in M1 liquid medium supplemented with 2 % (w/v) urea, 0.1 % (w/v) glucose and 0.002 % (w/v) phenol red for 7 days at 32 °C in 24-well plates under orbital shaking at 100 r.p.m. Acid production (red to yellow) is indicative for a urease negative result, while base production (red to orange) is indicative for a urease positive result. The oxidation-fermentation test was essentially performed as described by Hugh and Leifson [25] to test for fermentative metabolism of the relevant sugars. Test tubes with 4 ml M1 media, supplemented with 0.003 % Bromothymol blue, 0.3 % (w/v) agar and 0.2 % (w/v) glucose and 0.2 % (w/v) N-acetyl glucosamine, respectively, were inoculated with MBLW1^T^ and *G. obscuriglobus* and incubated for 6 days at 32 °C in the dark under oxidative and fermentative conditions (addition of a layer of mineral oil). In addition to the Hugh-Leifson test, carbohydrate utilization of MBLW1^T^ and *G. obscuriglobus* was determined on M1 agar plates supplemented with 0.2 % (w/v) of different carbohydrates after cultivation for 6 days at 32 °C.

Carbohydrate utilization profiles of *G. obscuriglobus* and *Z. formosa* are similar, with no apparent difference regarding the two main preferred carbohydrates, N-acetylglucosamine and glucose [5]. In the Hugh-Leifson test, both MBLW1^T^ and *G. obscuriglobus* display fermentative metabolism of N-acetylglucosamine and glucose (Table 1). Hence MBLW1^T^ grows best under aerobic conditions with carbohydrates, but displays similar facultative aerobic characteristics to *Schlesneria paludicola* [4] and *Planctopirus limnophila* [8, 26]. Further investigation of carbohydrate utilization showed similar profiles, except that MBLW1^T^, in contrast to *G. obscuriglobus*, does not grow on sucrose. Strain MBLW1^T^ displays additional distinct biochemical characteristics compared to *G. obscuriglobus*. MBLW1^T^ is catalase negative, oxidase positive and urease positive, while *G. obscuriglobus* is catalase positive, oxidase negative and urease negative. *Z. formosa* has been reported to be catalase positive, oxidase positive and urease negative [5].

Analyses of fatty acid methyl esters (FAME) in strain MBLW1^T^ and the reference strain *G. obscuriglobus* were performed with aid from the Identification Service of the DSMZ, Braunschweig, Germany. Prior to FAME analysis, MBLW1^T^ and *G. obscuriglobus* were cultivated on M1 agar plates for 5 days at 32 °C, harvested, washed in 50 mM Tris, pH 7.5 and dried in a Speedvac at 45 °C.

The most abundant fatty acids in MBLW1T were 16 : 1 ω5c, 16 : 0 and 18 : 0, while the most abundant fatty acids in *G. obscuriglobus* were 18 : 0, 16 : 1 ω5c and 15 : 0 ANTEISO (Table 2). Three fatty acids (16 : 0 3OH, 14 : 0, 13 : 0 iso 3OH) were found with low abundance in MBLW1^T^ that were not identified in *G. obscuriglobus*. MBLW1^T^ displays therefore a distinct fatty acid profile compared to *G. obscuriglobus.* Furthermore, the fatty acid profile of MBLW1^T^ differs from *T. sphagniphila* and *Z. formosa*, obtained by phospholipid fatty acid analysis [5, 6], where the major fatty acids are 16 : 1 ω5c, 18 : 1 ω5c and 18 : 0. Fatty acids with relative abundance of less than 1 % are shown in Table S1.

**Table 2.** Fatty acid composition of MBLW1^T^ in comparison to *G. obscuriglobus* based on FAME analysis Values are percentages of total fatty acids. The major fatty acids of each species are highlighted in bold. Only fatty acids with a relative abundance of more than 1% are shown. A detailed summary of the fatty acid analysis is shown in Table S1.

Fatty acid	MBLW1^T^	*G. obscuriglobus*
15 : 0 ANTEISO	nd	**7.9**
16 : 0	**25.0**	3.6
16 : 0 ISO	nd	1.4
16 : 0 3OH	2.3	nd
16 : 1 ω5c	**59.0**	**27.5**
17 : 0	2.2	0.3
17 : 0 ANTEISO	nd	4.6
18 : 0	**6.7**	**49.2**
18 : 1 ω5c	2.5	2.5
18 : 1 ω7c	nd	1.6

The 16S rRNA gene sequence from MBLW1^T^ has been deposited in the EMBL/NCBI databases under accession number FJ811525.

The major characteristics that distinguish MBLW1^T^ from *G. obscuriglobus*, *T. sphagniphila* and *Z. formosa* are summarized in Table 1. Strain MBLW1^T^ differs from the type strains of related genera in the absence of motility, except for *T. sphagniphila*, and flagella, in the phylogenetic position based on 16S rRNA, in the absence of a *Gemmata*-group characteristic 10-base indel in 16S rRNA, several biochemical properties and in the fatty acid composition, including the presence of three fatty acids, C_14 : 0_, C_13 : 0_iso3OH and C_16 : 0_3OH. MBLW1^T^ shares with *G. obscuriglobus* the presence of a complex endomembrane system, spherical cell morphology and colony colour. We therefore propose strain MBLW1^T^ to be classified as the representative of a novel genus and species for which the name *Tuwongella immobilis* gen. nov., sp. nov. is proposed.

## Description of *Tuwongella* gen. nov.

*Tuwongella* (Tu.wong.el’la. L. dim. ending –*ella*; N.L. fem. n. *Tuwongella* referring to Tu-wong, a region of the Brisbane River, Australia).

Gram-negative cells are spherical, non-motile and reproduce by budding. Crateriform structures are visible over the entire cell surface. Cells do not have stalks and do not form rosettes. Colonies are translucent and pink. Mesophilic and neutrophilic cells are chemoorganotrophic facultative aerobes. The major fatty acids are 16 : 1 ω5 c and 16 : 0. Cells possess a complex endomembrane system characteristic for members of the phylum *Planctomycetes*. The genus is a member of the phylum *Planctomycetes*, order *Planctomycetales*, family *Planctomycetaceae*. The type species is *Tuwongella immobilis*.

## Description of *Tuwongella immobilis* sp. nov.

*Tuwongella immobilis* (im.mo'bi.lis. L. fem. adj. *immobilis* non-motile) referring to a non-motile member of the family *Planctomycetaceae*.

Displays the properties given in the genus description with following traits. Spherical cells have a diameter of 2.2–3.1 µm. Growth occurs at pH 6.0–10.5 (optimum at pH 7.5–8.0) and at temperatures of 20–40 °C (optimum at 32–36 °C). Growth is inhibited by more than 50 % at NaCl concentrations greater than 0.4 % (w/v). DNA G+C content is 57 mol%. Oxidase positive, catalase negative and urease positive. Carbon sources are N-acetylglucoseamine, arabinose, galactose, glucose, maltose and rhamnose. Does not utilize sucrose, fructose, mannitol, xylose and glycerol. Fermentative metabolism of N-acetylglucoseamine and glucose. Resistant to ampicillin, gentamicin and streptomycin. Sensitive to kanamycin and neomycin.

The type strain MBLW1^T^ (=CCUG 69661 ^T^=DSM 105045^T^) was isolated from the University Lake, on the St. Lucia campus of The University of Queensland, Brisbane, Queensland, Australia.

## Supplementary Data

996 Supplementary File 1
