# Supplementary material for: Tuwongella immobilis gen. nov., sp. nov., a novel non-motile bacterium within the phylum Planctomycetes
Source: Int J Syst Evol Microbiol. 2017 Oct 31;67(12):4923–9. doi: 10.1099/ijsem.0.002271 (PMC5845749; doi:10.1099/ijsem.0.002271)
Supplement: Supplementary File 1 [file ijsem-67-4923-s001.pdf]

SUPPLEMENTARY DATA

Journal: International Journal of Systematic and Evolutionary Microbiology

Title: *Tuwongella immobilis* gen. nov., sp. nov., a novel non-motile bacterium within the phylum *Planctomycetes*

Authors: Christian Seeger[1], Margaret K. Butler[2], Benjamin Yee[1], Mayank Mahajan[1], John A. Fuerst[3] and Siv G.E. Andersson[1,*]

Affiliations: [1]Department of Cell and Molecular Biology, *Molecular Evolution*, Uppsala University, Box 596, 751 24 Uppsala, Sweden

[2]Australian Center for Ecogenomics, School of Chemistry and Molecular Biosciences, University of Queensland, Brisbane, Queensland 4072, Australia

[3]School of Chemistry and Molecular Biosciences, University of Queensland, Brisbane, Queensland 4072, Australia

Corresponding author: *Email: siv.andersson@icm.uu.se; Phone: +46 (0)18-471 4379

Supplementary Figures

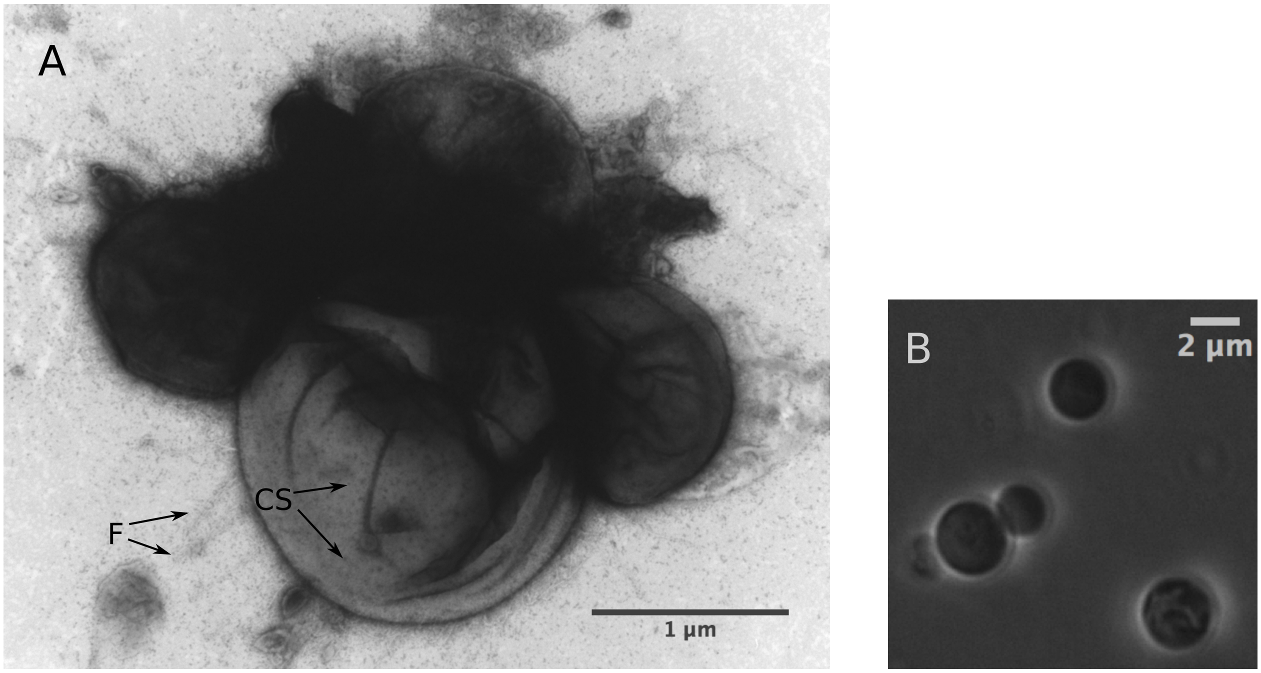

Supplementary Fig. 1. A) Transmission electron micrograph of negatively stained MBLW1^T shows crateriform structures (CS) distributed over the cell surface and fimbriae (F). **B**) Phase contrast micrograph of MBLW1^T shows absence of stalks and rosettes.

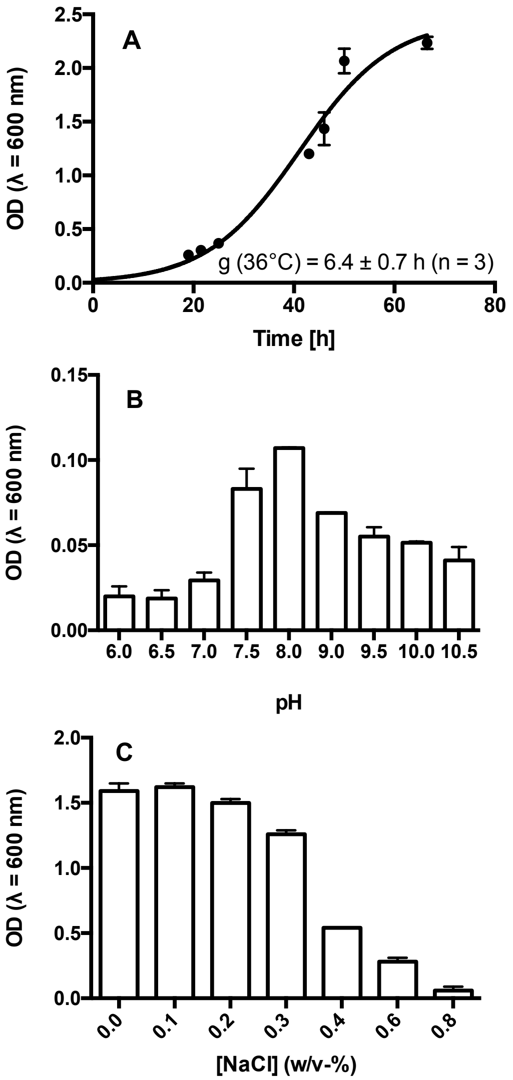

Supplementary Fig. 2. Biochemical and physiological characterization of MBLW1^T. **A**) Growth of MBLW1^T in M1 medium at 36°C was measured by the increase in optical density (OD) at λ = 600 nm over time. The generation time of 6.5 h was determined by fitting a logistic growth model to the experimental data using GraphPad Prism 6. **B**) Growth measured as optical density at λ = 600 nm of MBLW1^T in M1 medium at different pH values after cultivation for 2 days at 32°C. C) Growth measured as optical density at λ = 600 nm of MBLW1^T as a function of NaCl concentration (w/v-%) after cultivation in M1 medium for 5 days at 30°C.

Supplementary Table

Supplementary Table 1. Fatty acid profile of MBLW1^T and *G. obscuriglobus* based on FAME analysis. Values are percentages of total sum of fatty acids. n.d., not detected.

Fatty acid	MBLW1^T	*G. obscuriglobus*
13:0 ISO 3OH	0.1	n.d.
14:0	0.9	n.d.
15:0	0.7	n.d.
15:0 ISO	n.d.	0.9
15:0 ISO 3OH	n.d.	n.d.
15:0 ANTEISO	n.d.	7.9
16:0	25.0	3.6
16:0 ISO	n.d.	1.4
16:0 3OH	2.3	n.d.
16:0 ISO 3OH	n.d.	n.d.
16:1 ω5c	59.0	27.5
17:0	2.2	0.3
17:0 ISO	0.5	n.d.
17:0 ANTEISO	n.d.	4.6
18:0	6.7	49.2
18:1 ω5c	2.5	2.5
18:1 ω7c	n.d.	1.6
